# The sympathetic nervous system in healthy and hypertensive pregnancies: physiology or pathology?

**DOI:** 10.1113/EP089665

**Published:** 2022-12-02

**Authors:** Áine Brislane, Margie H. Davenport, Craig D. Steinback

**Affiliations:** ^1^ Program for Pregnancy & Postpartum Health Neurovascular Health Lab, Faculty of Kinesiology, Sport, and Recreation Women and Children's Health Research Institute Alberta Diabetes Institute University of Alberta Alberta Canada

**Keywords:** adrenergic control, muscle sympathetic nerve activity, pregnancy

## Abstract

The progression from conception through to the postpartum period represents an extraordinary period of physiological adaptation in the mother to support the growth and development of the fetus. Healthy, normotensive human pregnancies are associated with striking increases in both plasma volume and sympathetic nerve activity, yet normal or reduced blood pressure; it represents a unique period of apparent healthy sympathetic hyperactivity. However, how this normal blood pressure is achieved in the face of sympathoexcitation, and the mechanisms responsible for this increased activity are unclear. Importantly, sympathetic activation has been implicated in hypertensive pregnancy disorders – the leading causes of maternal–fetal morbidity and mortality in the developed world. An understudied link between pregnancy and the development of maternal hypertension may lie in the sympathetic nervous system regulation of blood pressure. This brief review presents the latest data on sympathoexcitation in both healthy and hypertensive pregnancies, and concurrent adaptations along the neurovascular cascade.

## Abstract (highlights)

- **What is the topic of this review?**
  Sympathoexcitation in both healthy and hypertensive pregnancies, and concurrent adaptations along the neurovascular cascade.
- **What advances does it highlight?**
  Known and plausible adaptations along the neurovascular cascade which may offset elevated MSNA in normotensive pregnancy while also highlighting knowledge gaps regarding understudied pathways.

## 1 INTRODUCTION

**FIGURE 1:**
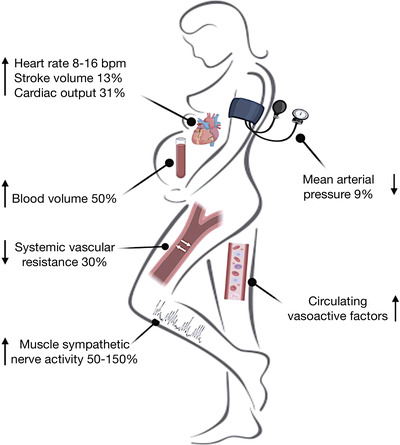
Systemic cardiovascular changes that arise in pregnancy.

Pregnancy is a time of rapid and profound cardiovascular adaptations to accommodate a growing fetus. Over the course of ∼40 weeks, there is a rise in maternal blood volume by 50%, a 31% increase in cardiac output and at least a 30% reduction in peripheral vascular resistance (Capeless & Clapp, 1989; Meah et al., 2016). Further to this, heart rate increases by 8–16 beats/min and blood pressure decreases reaching a nadir in the second trimester, before returning to early/pre‐pregnancy values within the third trimester (Loerup et al., 2019; Meah et al., 2016) (Figure 1). These latter changes (to heart rate and blood pressure) are mediated by a multitude of vasoactive factors such as nitric oxide, relaxin and oestrogen; however, the contribution of the autonomic nervous system is beginning to emerge (Chapleau et al., 1995; Ekholm et al., 1994; Fu & Levine, 2009). Governed by the autonomic system, the sympathetic nervous system is a key regulator of cardiovascular homeostasis through a complex cascade of neurovascular signalling resulting in the regulation of blood pressure and blood flow.

While the sympathetic nervous system is responsible for several functions, it is muscle sympathetic nerve activity (MSNA) that is of particular interest where the cardiovascular system is concerned. This is because MSNA impacts the vasculature via a cascade of the aforementioned neurovascular signalling that begins at the central nervous system (CNS). This signalling arises following activation and modulation of the descending neural drive initiated by multiple reflexes (such as the arterial baroreflex). This neural drive stimulates the recruitment of efferent sympathetic neurons, the type of neuron that carries information from the CNS to the target tissue. These neurons release sympathetic neurotransmitters (i.e., noradrenaline and neuropeptide Y (NPY)) that bind to and activate adrenergic receptors on vascular smooth muscle cells. This cascade of events helps to regulate smooth muscle tone and blood pressure through the actions and interactions of neurotransmitters and their receptors (Tymko et al., 2021).

Given the profound changes that arise within the cardiovascular system in pregnancy to ensure optimal fetal growth, it is vital to understand the adaptation of the neurovascular cascade during this time. By developing a thorough understanding of the normal physiological process that unfold at the neurovascular level, we may be able to elucidate the aetiology of cardiovascular‐related complications in pregnancy (i.e., gestational hypertension, pre‐eclampsia, gestational diabetes), so that timely and effective therapeutic intervention strategies can be developed in order to protect maternal and fetal health. The aim of this review is to describe the known adaptations along the neurovascular cascade that occur during healthy and complicated pregnancy, and to discuss potential clinical implications.

## 2 MSNA: AN OVERVIEW

MSNA can be directly assessed in humans at the level of the neuron using the gold standard technique of microneurography whereby bursts of spontaneous MSNA are captured. This approach allows direct recordings of the postganglionic efferent sympathetic neuron activity innervating the vascular smooth muscle within skeletal muscle (White et al., 2015).

The importance of measuring this outcome is supported by consistent findings in non‐pregnancy literature demonstrating the positive association between elevated MSNA, cardiovascular disease and associated risk factors. These include but are not limited to hypertension (Esler, 2000; Valensi, 2021), obesity (Grassi et al., 2000; Powell‐Wiley et al., 2021) and sleep apnoea (Somers et al., 1995; Tamisier et al., 2015; Taylor et al., 2016; Yeghiazarians et al., 2021). Specific to pregnancy, sympathetic neurovascular control is inherently heightened because of the dramatic adaptation of the cardiovascular system. Where pregnancy is coupled with cardiovascular risk factors such as those mentioned above, this neurovascular control is plausibly exacerbated further (Meah et al., 2016). The following sections describes the known MSNA response in uncomplicated and complicated pregnancies.

## 3 MSNA IN UNCOMPLICATED PREGNANCIES

Pregnancy is one of the only known ‘healthy’ physiological states of basal sympathetic hyperactivity (Jarvis et al., 2012). Augmented MSNA is thought to occur to help regulate blood pressure and blood flow in the face of pregnancy‐related reductions in vascular resistance (Usselman et al., 2015). The earliest microneurography recording in pregnancy was captured by Merrill and colleagues in 1995, illustrating for the first time that basal MSNA is indeed elevated in pregnancy compared with non‐pregnant controls (Merrill et al., 1995). Over the subsequent three decades, additional studies in healthy and complex pregnancies have been performed and these have been synthesised in a recent meta‐analysis.

During uncomplicated pregnancies, MSNA burst frequency (i.e., the number of bursts that arise per minute) is elevated at each trimester compared to the non‐pregnant state. Similarly, burst incidence is also elevated, which accounts for the progressive rise in heart rate that occurs in pregnancy (Meah et al., 2016). Limited longitudinal data support these meta‐analysed findings whereby MSNA is increased across pregnancy, beginning as early as the first trimester and maintained (or further elevated) in subsequent trimesters (Badrov et al., 2019; S. L. Hissen et al., 2017; Jarvis et al., 2012; Okada et al., 2015; Reyes, Usselman, Skow et al., 2018), before returning to baseline within 6 weeks of delivery (Fischer et al., 2004; Greenwood et al., 2001, 1998). Total MSNA, which encompasses burst frequency and burst amplitude, is also higher in pregnancy but unlike burst frequency and incidence, is higher in late versus early pregnancy, a response that is likely driven by augmented burst amplitude. Mechanistic insight into MSNA signalling has elucidated that sympathoexcitation is attributed to an increased number of integrated bursts of activity, coupled with increased firing from a given neuron (Schmidt et al., 2018). This suggests that increased MSNA in pregnancy may encompass both multi‐ and single‐unit hyperactivity (Greenwood et al., 2003, 1998). It is further plausible that with elevated neuronal discharge rates, downstream neurotransmitter release may be augmented during pregnancy (Huidobro‐Toro & Donoso, 2004; Macarthur et al., 2011).

## 4 MSNA IN COMPLICATED PREGNANCIES

Elevated MSNA is a consistent physiological characteristic of cardiovascular dysfunction in non‐pregnant populations as described earlier (Esler, 2000; Grassi et al., 2000; Maier et al., 2022; Powell‐Wiley et al., 2021; Somers et al., 1995; Tamisier et al., 2015; Taylor et al., 2016; Valensi, 2021; Yeghiazarians et al., 2021). The rise in basal MSNA in the face of cardiovascular disease is likely due to a combination of changes in central autonomic regulation, perturbation of the baroreflexes, altered body composition and sleep‐disturbed breathing (Joyner et al., 2008). It is, therefore, unsurprising that pregnancies complicated by obesity (S. Hissen et al., 2020; S. L. Hissen et al., 2022; Hsieh et al., 2020; Stickford et al., 2015), gestational hypertension (Badrov et al., 2019; Greenwood et al., 1998; Reyes, Usselman, Davenport et al., 2018) and obstructive sleep apnoea (Hsieh et al., 2020) all present with super‐elevated basal MSNA.

Early findings from Schobel and colleagues (1996) reported that pre‐eclampsia was also characterised by elevated MSNA when compared to healthy pregnancies. However, these findings are controversial since the authors failed to report augmented MSNA in healthy pregnancies compared to the non‐pregnant state, which is in opposition to all other data that has compared MSNA between pregnant and non‐pregnant individuals. With a seemingly anomalous comparator group, pre‐eclampsia was previously portrayed as a state of super‐elevated MSNA; a finding unsupported by subsequent investigations. For example, our group has shown that both burst frequency and incidence are comparable between pre‐eclamptic and uncomplicated pregnancies (Reyes et al., 2021). This is similar to the results of Foltmar‐Sander (2003), where pre‐eclampsia was not associated with exacerbated MSNA compared to normotensive pregnancy. While exploring the aetiology of pre‐eclampsia and gestational hypertension onset is beyond the scope of this review, it is necessary to acknowledge that these conditions are distinctly different from one another. Indeed, pre‐eclampsia has recently been described as a multi‐organ disease that when once manifested, elevated blood pressure is just one symptom. While many of the drug treatments for preeclampsia and gestational hypertension target the autonomic nervous system (i.e., labetalol, nifedipine, methyldopa) (Odigboegwu et al., 2018), unlike gestational hypertension pre‐eclampsia manifests at the placental level with potential for multiorgan dysfunction (Chappell et al., 2021). Therefore, while both pre‐eclampsia and gestational hypertension present with elevated blood pressure, it cannot be assumed based on this alone that both impact the sympathetic nervous system in a similar way. As such, there is an obvious need for this area of research to assess MSNA in both hypertensive and pre‐eclamptic individuals to truly understand how the sympathetic nervous system is impacted by these conditions and to help identify specific drug therapies for each condition.

## 5 NEUROVASCULAR TRANSDUCTION

Neurovascular transduction represents the functional effect of a given amount of sympathetic activity on diameter, stiffness and/or resistance within peripheral vessels (Steinback et al., 2019; Tymko et al., 2021). Neurovascular transduction can therefore be represented as peripheral vascular resistance, heart rate and blood pressure responses. Since pregnancy is already characterised by significant systemic vascular adaptations, including vasodilatation and reduced vascular resistance (Iacobaeus et al., 2016), the role of MSNA signalling on the vascular system is likely significant and warrants greater understanding (Boeldt & Bird, 2017).

Differences in neurovascular transduction between the pregnant and non‐pregnant state were first highlighted by Jarvis et al. (2012). The authors assessed neurovascular transduction by quantifying the relationship between forearm vascular resistance and MSNA parameters, including total activity and bursts frequency, to a head‐up tilt protocol. Despite similar blood pressure and heart rate responses between groups, neurovascular transduction was blunted in pregnancy compared to pre‐pregnancy when forearm vascular resistance was expressed relative to total activity or burst frequency. In agreement with Jarvis and colleagues, our laboratory observed blunted neurovascular transduction evidenced by lower total peripheral resistance (calculated as mean arterial pressure/cardiac output), and maintenance of lower‐to‐normal blood pressure despite elevated MSNA in pregnancy compared to the non‐pregnant state. This indicates an offsetting of neurovascular control in uncomplicated pregnancies, echoing data from Usselman et al. (2015). Interestingly, Usselman and colleagues identified strong positive relationships between peripheral vascular resistance and arterial blood pressure during a cold stress challenge in non‐pregnant women, but not in uncomplicated pregnancy (Usselman et al., 2015). Steinback et al. (2019) examined neurovascular transduction further and identified that in response to individual or sequences of resting sympathetic activity, mean arterial pressure is lower and R–R intervals (i.e., the duration of the cardiac cycle) are smaller in pregnant versus non‐pregnant controls. These summative data strongly support the concept that neurovascular transduction is blunted in pregnancy.

**FIGURE 2:**
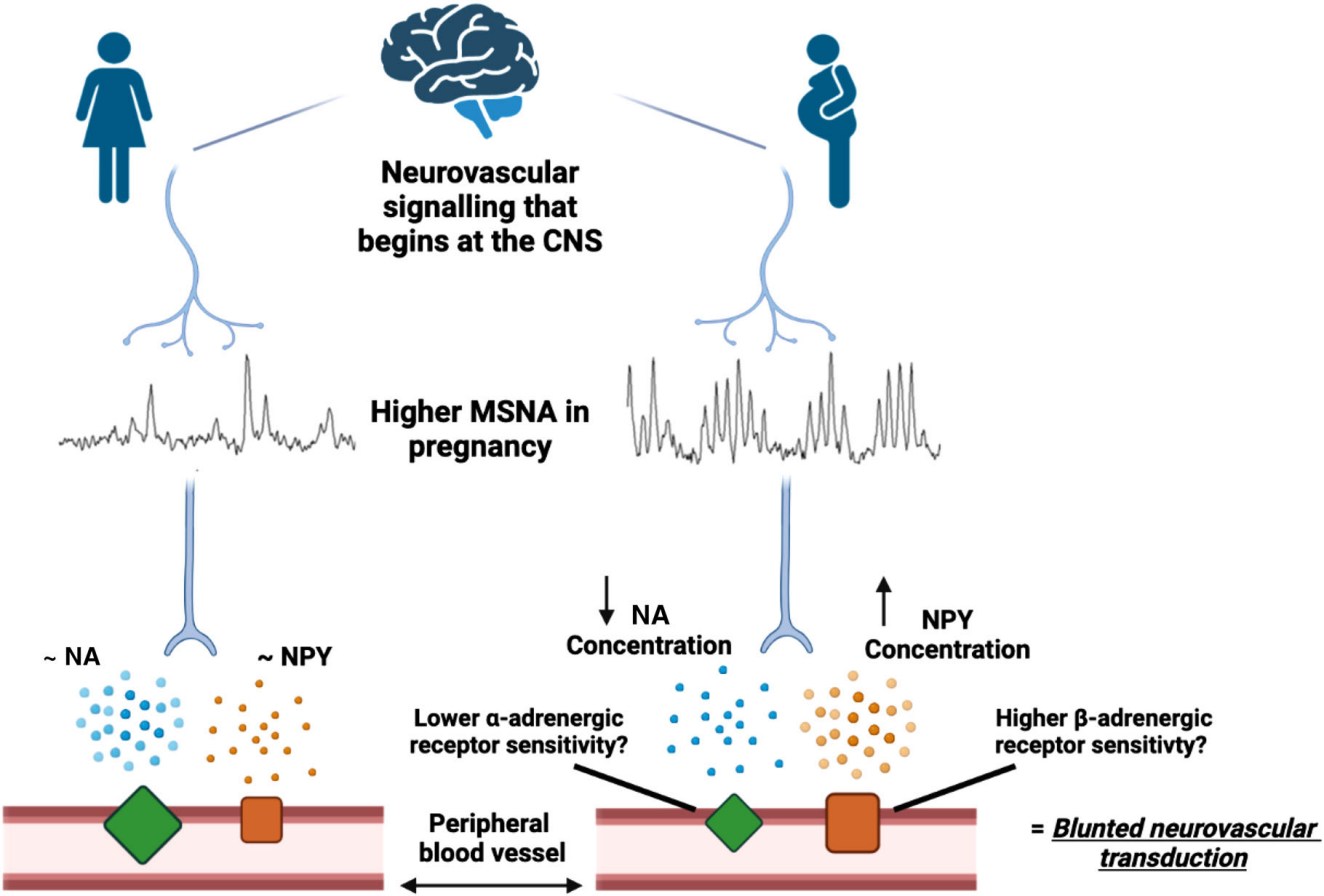
Schematic representation of plausible mechanisms at peripheral blood vessels underpinning blunted neurovascular transduction in pregnancy compared to the non‐pregnant state. NA, noradrenaline; NPY, neuropeptide Y.

The reasons for blunted neurovascular transduction are unknown (Figure 2), and it has yet to be determined if it is a pregnancy‐specific adaptation or a response to elevated MSNA. It is plausible that the neurovascular signalling cascade adapts to elevated sympathetic activity to maintain arterial pressure in normotensive pregnancies and, therefore, neurovascular blunting may be perceived as a positive adaptation.

## 6 MECHANISMS OF BLUNTED NEUROVASCULAR TRANSDUCTION

The mechanisms underpinning blunted neurovascular transduction in pregnancy have yet to be fully understood. However, they may pertain to elevated nitric oxide levels, increased receptor density for vasodilators, decrease and increase in α_1_‐ and β_1_‐adrenergic receptor sensitivity and/or density, respectively, or reduced concentrations of signalling molecules within the neurovascular junction (Schmidt et al., 2018; Steinback et al., 2019). Herein, we describe the potential adaptation of neurotransmitters and their receptors that may elucidate pregnancy‐related neurovascular transduction further.

## 7 CIRCULATING NEUROTRANSMITTERS

Neurotransmitters carry information between neurons and target tissues (Hyman, 2005) and while it is beyond the scope of this review to explore all of them, we include what is known in relation to pregnancy. Noradrenaline is one of the main neurotransmitters released from postganglionic sympathetic neurons in peripheral tissues. In the periphery, it increases arterial load, modifies vascular tone, stimulates vasoconstriction and, in turn, increases blood pressure (Schroeder & Jordan, 2012). Circulating concentrations of noradrenaline are dependent on its release, binding, re‐uptake, and specific to pregnancy, blood flow and plasma volume. The latter two are of particular interest since they both become elevated during pregnancy (Aguree & Gernand, 2019; Thaler et al., 1990). Therefore, the relationship between pregnancy‐mediated increases in MSNA and circulating noradrenaline is likely complex and multi‐factorial.

Cross‐sectional and longitudinal data from Tunbridge and colleagues has shown that across pregnancy, plasma noradrenaline concentrations are reduced, likely a consequence of the known plasma volume expansion during this time (Aguree & Gernand, 2019; Tunbridge & Donnai, 1981). Longitudinal data have also reported noradrenaline concentrations to be elevated in early‐ and reduced by late‐pregnancy (Okada et al., 2015), the latter of which has been reported from Kaaja et al. (2004) and (Natrajan et al., 1982). Together, this implies that as pregnancy progresses, fewer neurotransmitters, at least where noradrenaline is concerned, are in the circulatory system, which may be in response to or a resultant of elevated MSNA to maintain vascular homeostasis.

NPY is a potent vasoconstrictor peptide that is transmitted with noradrenaline at sympathetic nerve terminals after sympathetic stimulation (Morris et al., 1986; Wahlestedt et al., 1990). According to cross‐sectional data, NPY concentrations are higher in pregnancy compared to the non‐pregnant state (Khatun et al., 2000). This is apparent in the first trimester, and sustained into late‐pregnancy (Petraglia et al., 1989). NPY is found in non‐adrenergic neurons that act on arteries, including the uterine artery, and therefore is an important regulator of uterine blood flow. In pregnancy, there is an increase in the number of NPY‐containing nerve fibres supplying the uterine artery, positioning NPY as the main vasoconstrictor of the uterine artery (Mione et al., 1990; Stennett & Khalil, 2006). This pregnancy‐mediated increase in NPY concentrations is even higher for individuals with pre‐eclampsia and eclampsia (Khatun et al., 2000). Given its role in dominating uterine artery blood flow, NPY may be a critical neurotransmitter that is pivotal to fetal development (Jovanovic et al., 2000). To achieve this blood flow regulation, it is plausible that NPY may interact with other vasoactive agents by decreasing the release of vasodilators at the vascular endothelium (Grundemar & Högestätt, 1992), increasing vascular smooth muscle Ca^2+^ (Cressier et al., 1995), reducing cAMP production and/or membrane depolarisation (Prieto et al., 1997). These actions in turn likely influence neurovascular transduction and may in part explain blunted responses in pregnancy compared with the non‐pregnant state.

## 8 NEUROTRANSMITTER RECEPTOR ADAPTATION

Peripheral neurovascular transduction may be blunted in pregnancy because of a decrease in α‐adrenergic receptor density and/or sensitivity. Nisell and colleagues demonstrated that the change in systemic, although not calf, vascular resistance in response to noradrenaline infusion was significantly less in pregnancy compared with non‐pregnant controls (Nisell et al., 1985). The authors speculated that the observed responses may be due to a reduced sensitivity to α_1_‐adrenoreceptors (Nisell et al., 1985); however, it appears that this may be dependent on the vascular bed in question. In line with this, at peripheral vascular sites where α_1_‐receptor sensitivity is increased compared to the non‐pregnant state, it may be countered by increased β_2_‐adrenergic sensitivity, thus blunting neurovascular transduction (Reyes, Usselman, Davenport et al., 2018). Evidently, neurovascular transduction is a complex interplay between MSNA signalling, circulating neurotransmitters and associated receptors at the vascular beds, and our understanding of this neurovascular cascade is only in its infancy relative to human pregnancy.

To elucidate the mechanisms underpinning neurovascular transduction in pregnancy, future research should aim to quantify neurotransmitter concentrations alongside other outcomes of sympathetic activity such as MSNA. To create a holistic picture of neurovascular transduction in pregnancy, these outcomes should be measured at rest and during sympathetic activation in normotensive and complicated pregnancies. In doing so, we may be able to identify markers of sympathetic maladaptation in pregnancy and thus, enhance our capacity to predict cardiovascular‐related complications (such as gestational hypertension), that have become recognised as a time of exacerbated MSNA.

## 9 CONCLUSION

During an uncomplicated pregnancy, MSNA is increased, and this is further exacerbated for some but not all pregnancy complications. Despite elevated MSNA, neurovascular transduction is reduced in healthy pregnancy and thought to be attributed to a reduced concentration of circulating neurotransmitters as well as reduced receptor sensitivity. There is a great need for future work in this field to quantify neurotransmitters and sensitivity alongside sympathetic activity to help explain the relationship between increased sympathetic outflow and the end‐organ vascular response.

## AUTHOR CONTRIBUTIONS

Áine Brislane, Craig D. Steinback and Margie H. Davenport: design of the work, interpretation of data for the work, drafting of and critically revising the work for important intellectual content. All authors have read and approved the final version of this manuscript and agree to be accountable for all aspects of the work in ensuring that questions related to the accuracy or integrity of any part of the work are appropriately investigated and resolved. All persons designated as authors qualify for authorship, and all those who qualify for authorship are listed.

## CONFLICT OF INTEREST

None.
